# Non-invasive scalp recording of electroencephalograms and evoked potentials in unanesthetized horses using a 12-channel active electrode array

**DOI:** 10.3389/fvets.2024.1470039

**Published:** 2024-12-02

**Authors:** Kosuke Itoh, Norihide Kikumura, Tamao Maeda, Satoshi Hirata, Monamie Ringhofer

**Affiliations:** ^1^Center for Integrated Human Brain Science, Brain Research Institute, Niigata University, Niigata, Japan; ^2^Faculty of Life and Environmental Sciences, Teikyo University of Science, Uenohara, Japan; ^3^Wildlife Research Center, Kyoto University, Kyoto, Japan; ^4^Research Center for Integrative Evolutionary Science, The Graduate University of Advanced Science (SOKENDAI), Hayama, Japan

**Keywords:** equine, EEG, auditory evoked potential (AEP), animal welfare, neuroimaging

## Abstract

Despite the long history of the horse-human bond, our understanding of the brain and mind of horses remains limited due to the lack of methods to investigate their brain functions. This study introduces a novel methodology for completely non-invasive, multi-channel recording of electroencephalography (EEG) and evoked potentials in awake horses to examine equine auditory cortical processing. The new approach utilizes specially designed brush-shaped active electrodes that facilitate stable signal acquisition through the hair coat by penetrating electrode pins and integrated pre-amplifiers. A 12-channel electrode array provided greater scalp coverage than prior work. As a proof of concept, clear cortical auditory evoked potentials (CAEPs) were recorded in response to sound onsets and offsets. The equine CAEP waveform morphology resembled the human P1-N1-P2-N2 complex, although the latencies were shorter than typical human values. The CAEP amplitudes were maximal at centroparietal electrodes, contrasting with the frontocentral distribution seen in humans, potentially explained by differences in auditory cortex orientation between species. This non-invasive multi-electrode method enables the evaluation of cognitive abilities, normal and abnormal brain functions, and advances scientific understanding of the equine mind. It offers potential widespread applications for recording EEGs and evoked potentials in awake horses and other medium-to-large mammalian species.

## 1 Introduction

The bond between humans and horses has evolved over millennia, culminating in the current practices of utilizing horses for transportation, recreation, competitive sports, and therapy. To sustain and enrich this human-equine relationship, a scientific understanding of the equine mind and brain is essential. However, limited knowledge exists concerning the higher brain functions of horses and their associated disorders (1, 2), reflecting the inherent challenges of conducting laboratory experiments with these animals.

Scalp-recorded cortical evoked potentials (EPs) and event-related potentials elicited by sensory stimuli, such as auditory, visual, and somatosensory stimuli, serve as valuable tools in human medicine and neuroscience for non-invasively probing cerebral functions and associated disorders. However, the utilization of scalp-recorded cortical EPs in equine subjects has been scarce, despite their potential benefits. This underutilization stems primarily from methodological challenges specific to recording cortical EPs in alert horses, rather than a general difficulty in acquiring electrical signals from the living equine brain. Prior studies have successfully recorded continuous electroencephalograms (EEGs) in conscious horses (3–6), as well as brainstem auditory (7, 8), visual (9, 10), and somatosensory (11, 12) evoked potentials under anesthesia. Nonetheless, equine recordings of cortical EPs without sedation remain sparse, to our knowledge, with only one study reporting the acquisition of visual evoked potentials in non-sedated horses using invasive subcutaneous needle electrodes (13). The lack of non-invasive methods for awake cortical EP is not specific to horses but is a common issue for other medium to large-sized mammals, such as such cattle and swine (14–17), although successful attempts have been made to record non-invasive EEG in smaller animals, such as dogs (18) and monkeys (19, 20).

The primary objective of this study was to develop a methodology for completely non-invasive recording of cortical EPs in unanesthetized equine subjects. This development was an extension of our previous success on recording non-invasive EEG and EP in smaller animals, namely, macaque monkeys and common marmosets (19–21). We focused specifically on the auditory modality to record cortical auditory evoked potentials (CAEPs) in horses, although the methodology developed herein would be widely applicable for acquiring various types of EPs and continuous EEG in horses and other medium to large-sized mammals, such as cattle and swine.

The secondary objective was to characterize the morphology and scalp topography of scalp-recorded CAEPs in equines, thereby identifying the homologous components to those observed in human CAEPs (e.g., P1, N1, and P2). This would facilitate future utilization of equine CAEPs for neurological assessments and scientific investigations into equine auditory processing.

To achieve these objectives, it was necessary to employ a sufficient number of EEG electrodes that provided extensive coverage over the areas of the head surrounding the animal's brain. This necessity arises from the fact that EEG and evoked potential recordings reflect the voltage differential between pairs of scalp electrodes, representing the summation of volume-conducted electrical activity originating from the brain. Volume conduction refers to the phenomenon wherein electrical signals generated by neural activity propagate through the head tissues (including the brain, skull, and skin), resulting in recorded potentials that are spatially smoothed and distorted representations of the underlying neural sources. In humans, for instance, CAEPs exhibit maximal amplitudes over frontocentral scalp regions, as electric currents originating in the left and right auditory cortices on the lower banks of the lateral sulci project upwards to converge around the vertex (22). Conversely, the scalp topography of equine CAEPs remains unknown, as the precise location of the horse auditory cortex has not been determined, and the effects of volume conduction in this species are also uncharacterized.

Therefore, an exploratory investigation utilizing a multi-electrode array to provide broad coverage across the equine scalp was requisite to empirically determine the topography of CAEPs in horses. This posed a technical challenge, as the dense hair coat of the equine head, which has high electrical impedance, hinders reliable multichannel acquisition of EEG signals from the scalp. This is particularly true when the horse is alert and moves its head, destabilizing the skin-electrode contact. To our knowledge, previous non-invasive EEG and EP recordings in conscious horses have been limited to a maximum of four frontal electrodes, although the number of electrodes could be increased to nine when sedated (9), or 11 in a partly invasive awake recording wherein the hair was shaved and the exposed skin was abraded with sandpaper to reduce electric impedance (3).

**Figure 1:**
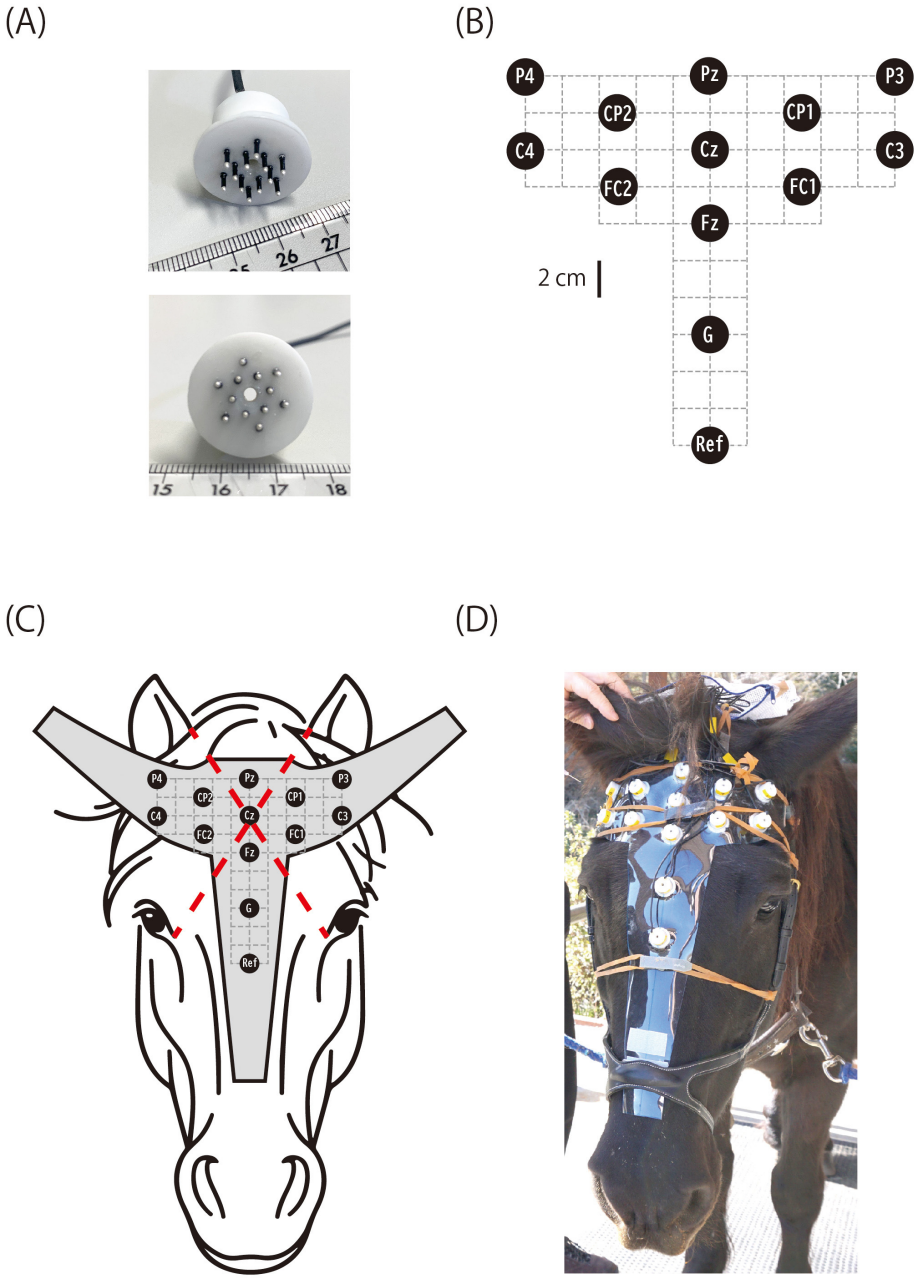
Horse EEG electrode and layout. **(A)** Brush-shaped active electrode with pins that penetrate the hair coat. The cylindrical housing contains a pre-amplifier. **(B)** Electrode layout (G, ground; Ref, reference). **(C)** The electrodes were fixed on a vinyl sheet cut to the shape of a horse's head, and the position of Cz was determined as the intersection of two lines, each connecting the inner corner of one eye and the medial rim of the contralateral ear. **(D)** Horse C wearing the electrode cap.

To address this challenge, we introduced a novel animal EEG recording methodology utilizing specially designed brush-shaped, active electrodes (Figure 1). A brush-shaped electrode facilitates signal acquisition via silver pins that partly penetrate through the hair coat, thereby enabling a more stable electrical interface between the skin and electrode compared to conventional disk electrodes, without necessitating hair removal. Additionally, these electrodes were “active,” incorporating small pre-amplifiers to facilitate low-noise recordings even when electrode-skin impedances could not be minimized. Active electrodes are becoming increasingly prevalent in human EEG applications such as brain-computer interfaces and infant recordings, and this study is the first to apply this device to equine EEG recording, to our knowledge. As a proof of concept, we successfully recorded completely non-invasive CAEPs in three awake equine subjects using a 12-channel electrode array providing more extensive scalp coverage than previously achieved.

## 2 Materials and methods

### 2.1 Subjects

Three equine subjects (Equus ferus caballus) were utilized, referred to as Horses A, B, and C. Horse A was a 10-year-old Thoroughbred gelding with an estimated weight of 520 kg. Horse B was a 23-year-old Halflinger gelding weighing ~420 kg. Horse C was a 28-year-old crossbred mare with an estimated weight of 470 kg. Horse A was housed at Niigata University, while Horses B and C were maintained by Teikyo University of Science. All experimental procedures were approved by the Internal Review Board of Niigata University.

### 2.2 Stimuli

Pure tones with frequencies of 1,200 and 1,500 Hz were presented in an oddball paradigm. We chose these frequencies to ensure they are within the hearing ranges of horses (23), humans, and non-human primates (24, 25), thereby enabling future comparisons of CAEPs across species. Stimulus duration was 300 ms, with sound onset asynchronies randomly varying between 700 and 900 ms. The standard stimulus was presented on 80% of trials, while the deviant stimulus occurred on the remaining 20%. Stimuli were delivered in uninterrupted blocks of 300 trials lasting 4 min, with standard and deviant tones counterbalanced across blocks: the 1,200 Hz tone served as the standard in half the blocks, and 1,500 Hz as the standard in the other half. The number of blocks was determined by confirming CAEP waveforms. Block counts varied between subjects due to inconsistent signal-to-noise ratios across horses and recording sessions. For Horse A, 56 blocks of data (16,800 trials) were collected over 7 days; for Horse B, 38 blocks (11,400 trials) were collected over 3 days; and for Horse C, eight blocks (2,400 trials) were collected in 1 day.

The stimuli sequences were digitally synthesized on-the-fly using LabVIEW (26) on a notebook computer (Latitude 5330, Dell Technologies), converted to analog signal by an analog-to-digital converter (NI-9262, National Instruments), amplified, and delivered via a powered loudspeaker (MS101 II, Yamaha Corporation) positioned ~1.5 m from the subject's head. Digital triggers informing sound onset timing were sent from LabVIEW via a digital input-output interface (NI-9401, National Instruments) to the EEG amplifier, delayed by 4.4 ms to accommodate the sound traveling time from the loudspeaker to the subject. Stimulus sound pressure level was ~60 dB SPL measured at the subject's head.

### 2.3 EEG electrode and montage

A novel brush-shaped active electrode (BA-EEG electrode, TK221-009) was designed and manufactured in collaboration with Unique Medical Co., Ltd. (Tokyo, Japan; Figure 1). The electrode consisted of 12 silver pins (1 mm diameter, 5 mm length) protruding from a cylindrical plastic casing that housed a preamplifier. All pins were electrically interconnected within the casing. The number and size of the pins were optimized to balance the electrode's mechanical durability with the stability of the skin-electrode contact. The preamplifier performed impedance transformation to reduce noise interference along the lead wire connecting to the main EEG amplifier. The plastic casing had a central hole (2 mm diameter) through which EEG gel could be applied after fixing the electrode on the horse's head. Although the electrode could sometimes be used dry when the hair was short, applying electrode gel significantly improved signal quality by ensuring stable electrical contact between the skin and electrode pins. Each electrode was connected via a 0.6 m lead wire to a battery-powered unit (TK219-013, Unique Medical Co., Ltd.) fixed to the horse's headcollar at the nape during recording. This unit supplied power to the preamplifier and relayed the EEG signal from the electrode to the main amplifier. Twelve active electrodes recorded EEGs, with one serving as the reference channel. Additionally, a passive electrode of the same shape but without a preamplifier circuit served as the ground channel. All electrodes were attached to a 1 mm thick vinyl sheet cut to fit the horse's head, constructing an electrode cap (Figure 1).

After extensive exploration using Horse A, a referential (monopolar) montage with the electrode layout shown in Figure 1C was deemed appropriate for recording equine CAEPs. The position of the central electrode (Cz) was defined as the intersection of two diagonals, each connecting the inner corner of one eye to the medial edge of the contralateral earlobe. The positions of the other electrodes were determined relative to Cz, as illustrated in Figure 1. The electrodes were labeled according to the International 10-10 system for electrode placement (27). However, due to the difficulty in identifying key anatomical landmarks required by the 10-10 system (e.g., nasion, inion, and the left and right preauricular points) on the horse head, the electrode names used here only approximated their intended positions based on the 10-10 nomenclature. The position of the reference electrode was slightly below the level of the eyes (Figure 1).

### 2.4 EEG recording

Horse A was acclimated to the headcollar by wearing it for 1 h per day over 4 days. In contrast, Horses B and C did not require an adaptation procedure, likely because they were accustomed to interacting with unfamiliar humans through activities such as horseback riding events.

The horses remained vigilant yet calm throughout the EEG recordings, which were conducted during daytime in their stables. For preparation, a headcollar was applied, and the electrode cap (Figure 1) was attached to the head by fixating it to the headcollar using Velcro. Light pressure was applied to press the electrodes toward the scalp by subtending rubber bands over and across the electrode cap, with their ends fixed to the cheekpieces of the headcollar on both sides. After positioning the electrode cap, 1 cm^3^ of electrode gel (V15, Brain Products, Germany) was applied to each electrode through its central hole (Figure 1) using a syringe with a blunted needle. The electrode leads were connected to the power unit, a plastic case fixed to the crownpiece of the headcollar at the nape. The power unit relayed the EEG signals from the electrodes to the main amplifier (Brain Amp DC, Brain Products, Germany) via a 3 m cable.

The EEGs were acquired with a 0.016–100 Hz bandpass filter and digitized at 5 kHz with 0.1 μV/bit precision for Horse A; the data were resampled to 1 kHz prior to analyses. For Horse C, the sampling rate was 1 kHz with a 0.016–100 Hz bandpass filter and 0.1 μV/bit precision. The amplifier setting for Horse B was the same as Horse C, except half the data were recorded with a 100 Hz lowpass filter without a lowcut for direct current recording.

### 2.5 CAEP analysis

The data were analyzed utilizing the EEGLAB software (28) with the ERPLAB extension (29). The continuous EEG data were bandpass filtered (2–40 Hz), segmented time-locked to the onset of the stimulus (−100 to 400 ms), corrected for baseline by subtracting the pre-stimulus period average, screened for artifacts, and rejected if they contained an artifact of ±100 μV relative to the baseline. Finally, the artifact-free segments were averaged to obtain cortical auditory evoked potentials (CAEPs). The number of non-rejected data segments was 15,971 (95.1%), 9,871 (86.6%), and 1,953 (81.4%) for Horses A, B, and C, respectively. Since no apparent and reliable differences were observed in the CAEPs between the standard and deviant stimuli of the oddball sequence, all trials were pooled together to obtain a single waveform of CAEP for each horse.

## 3 Results

### 3.1 Continuous EEG

**Figure 2:**
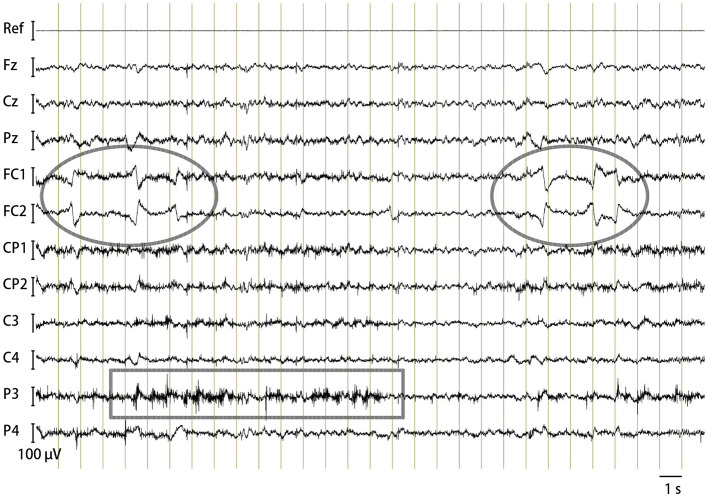
Horse EEG. An example trace of bandpass filtered (1–100 Hz) continuous EEG of Horse B containing typical artifacts: oval, eye movements; rectangle, electromyogram.

Noise-free EEGs were successfully recorded from alert but quiet horses, as shown in Figure 2. Typical artifacts included those caused by eye movements as observed concurrently with the EEG by the experimenter (Figure 2, marked with ovals), which were usually recorded at the FC1 and FC2 electrodes. This was reasonable because these electrode sites were near and tangential to the retinal plane, the major source of electric currents originating from the eye (30). Subtle muscular tensions that did not cause overt movements manifested as electromyograms (EMGs), mostly at posterior electrodes such as Pz, CP1, CP2, P3, and P4 (Figure 2, marked with a rectangle). This spatial feature could be explained by their anatomical positions relative to the underlying temporalis muscle. Gross head and body movements resulted in extensive artifacts over all electrodes, and such EEG segments were automatically discarded from the CAEP analyses. The EEG power spectrum of the horses, reflecting alpha, beta, and other activities, varies significantly among individuals and with the animals' arousal states (4–6). Analyzing the commonalities and variations in the continuous EEG power spectrum was beyond the scope of this study.

### 3.2 CAEP

**Figure 3:**
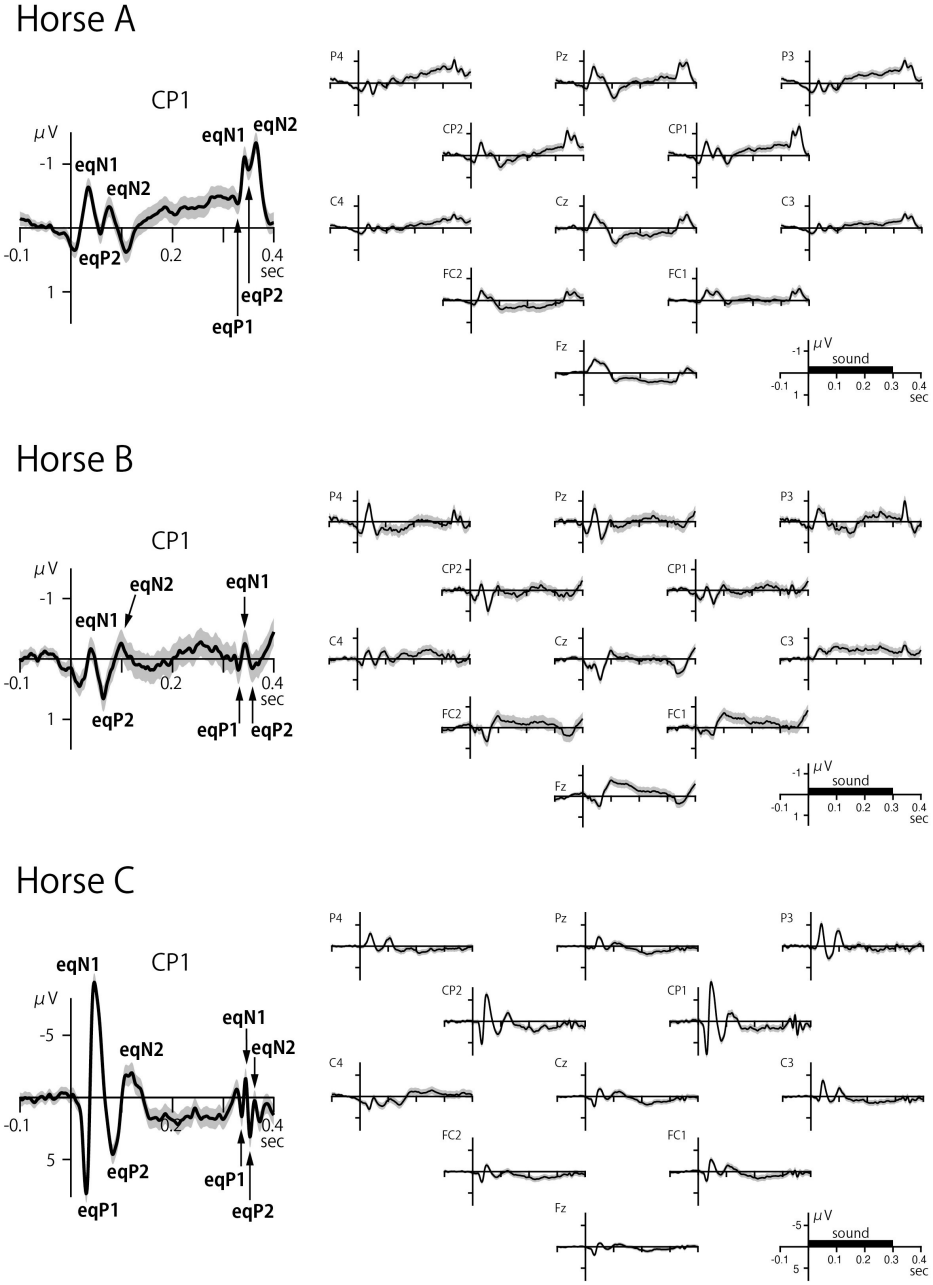
Scalp-recorded CAEPs in three horses. The left panel shows the CAEP at electrode CP1, and the right panel shows the waveforms for all electrodes. The onset and offset of sound elicited transient responses within a latency of 100 ms over distributed areas of the scalp. The black bar on the time axis indicates the duration of the sound stimulus (300 ms). The shaded error band represents 2 times the standard error.

Clear CAEPs were elicited by the onset of sounds, referred to as onset-CAEP, and to the offset of sounds, referred to as offset-CAEP, in all horses (Figure 3, Table 1). The overall features of waveform morphology and scalp topography of onset-CAEP and offset-CAEP were largely similar across subjects, although individual differences were observed.

**Table 1:** Peak latency and peak amplitude of horse CAEP components.

	**Onset CAEP**			
**Horse**	**eqP1**	**eqN1**	**eqP2**	**eqN2**
A	n.a.	35 ms	51 ms	71 ms
		−0.76 μV	0.50 μV	−0.42 μV
		(Pz)	(P4)	(FC1)
B	n.a.	41 ms	62 ms	100 ms
		−0.89 μV	1.01 μV	−0.77 μV
		(P4)	(Cz)	(Fz)
C	30 ms	46 ms	82 ms	97 ms
	7.77 μV	−9.28 μV	4.60 μV	−3.61 μV
	(CP1)	(CP1)	(CP1)	(P3)

The latencies of the CAEPs were measured at the electrode (indicated in parentheses) where the component displayed the maximum amplitude across the scalp.

n.a., not available.

Like the human onset-CAEP that typically has four exogenous components called P1, N1, P2, and N2 (32, 33), the equine onset-CAEP also comprised a series of positive-negative-positive-negative waves, although some components were not clearly identified in some cases (marked as n.a. in Table 1). These components were labeled here as eqP1, eqN1, eqP2, and eqN2, where “eq” stood for equine. The approximate peak latencies for these waves were 30 ms (eqP1), 40 ms (eqN1), 50–80 ms (eqP2), and 70–100 ms (eqN2) as summarized on Table 1. The peak latencies were measured at the electrode displaying the highest amplitude for the CAEP components.

The offset-CAEP was recorded as a wave complex that occurred 30–80 ms after sound termination. Based on polarity and latency, four components similar to those found in the onset-CAEP were identified: eqP1, eqN1, eqP2, and eqN2 (Table 1). The amplitudes of offset-CAEPs were apparently smaller than those of the onset-CAEP, similar to human findings (34).

**Figure 4:**
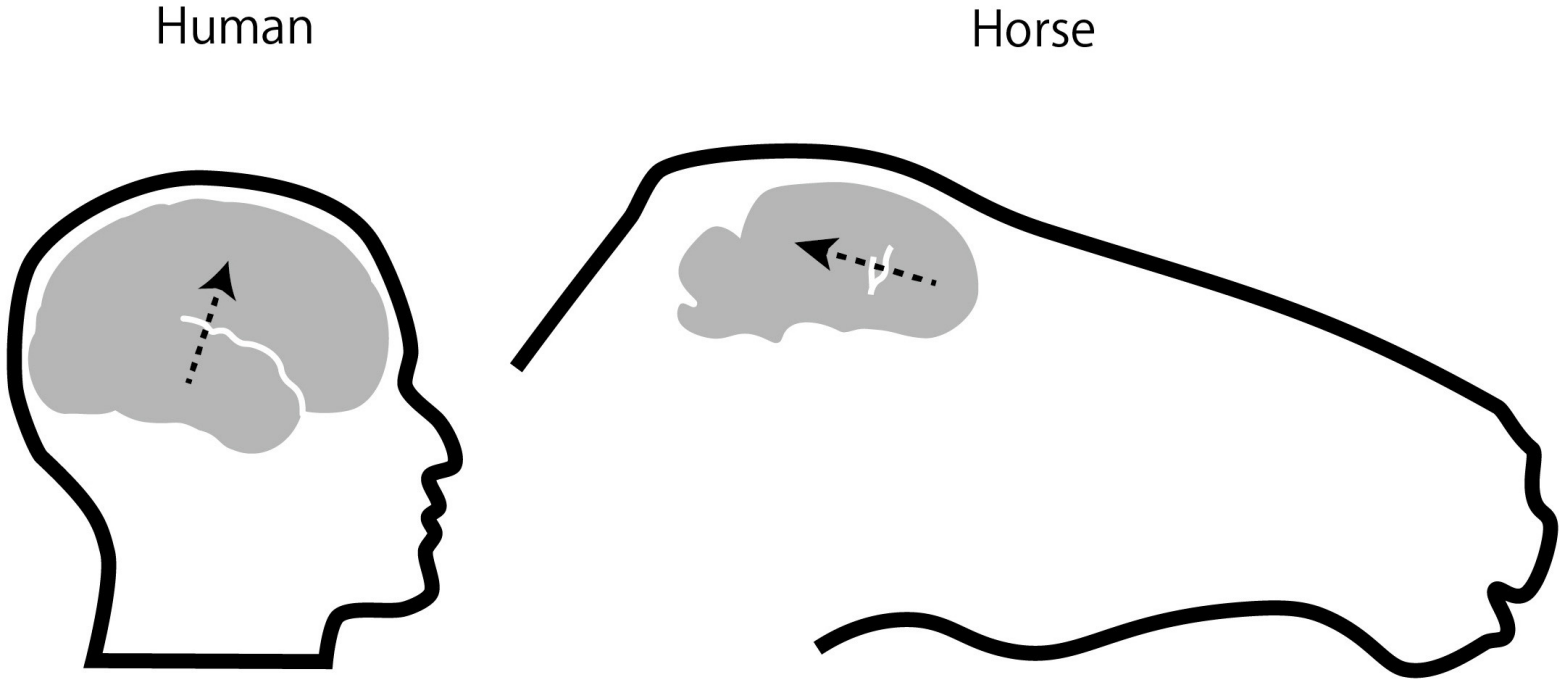
Position and orientation of EEG sources in auditory cortex. Assuming that the equine auditory cortex is located on the lower bank of lateral sulcus (also called Sylvian fissure, draw in white) as in humans, electric dipoles of auditory cortical activities in the horse would project posteriorly as opposed to fronto-centrally in humans (arrows). The equine brain and lateral sulcus were drawn based on Figure 6 in Johnson et al. (31).

The CAEP waveforms were broadly distributed over the scalp, consistent with volume conduction. Whereas human CAEPs usually have a frontocentral distribution when ear, mastoid, or nose reference is used, equine CAEPs showed the greatest amplitude over centroparietal electrode sites when the reference electrode was placed on the nose bridge below the eyes. This species difference might be explained by the orientation of the Sylvian fissure (lateral sulcus). In humans, the sulcus is oriented roughly horizontally with a slight downward tilt from the back to the front of the brain, causing the electric dipoles in the auditory cortex to project to the frontocentral scalp. In horses, the sulcus is more vertically oriented (31), causing the dipoles in the auditory cortex to project posteriorly (Figure 4). However, this interpretation assumes that the auditory cortex in horses is located on the lower bank of the Sylvian fissure, a hypothesis that, to our knowledge, has not been confirmed through studies of cortical cytoarchitecture or fiber connections. For instance, in the African wild dog, the cytoarchitectonically defined auditory cortex is situated outside and dorsal to the Sylvian fissure (35), which, like in horses, is oriented vertically. Further research is needed to explain the scalp topography of equine CAEP.

Regarding left-right asymmetry, the amplitudes were largely symmetric in Horse A when comparing the CAEP waveforms between corresponding electrodes across the hemispheres, such as P4 vs. P3 and CP1 vs. CP2 (Figure 3). In contrast, the eqN1 amplitude showed slight right dominance in Horse B when comparing P4 to P3, while in Horse C, it displayed slight left dominance when P4 and CP2 were compared to P3 and CP1, respectively. Intriguingly, in Horse C, the greatest CAEPs were recorded not at the midline but slightly laterally at CP1 and CP2. Such a topography is rarely, if ever, observed in neurologically normal human subjects. This topography in Horse C could be explained by assuming that the electric dipoles were oriented slightly laterally, reminiscent of the radial components of the human CAEP recorded over the temporal scalp (36). However, given the individual differences in scalp topography among the three horses, this issue warrants further investigation.

Another interesting feature of Horse C was that the CAEP amplitude was ~10 times greater than in the other two horses. Although the exact reason for this difference remains unclear, potential causes may include variations in skull and hair coat thickness, which can affect impedance.

## 4 Discussion

The use of innovative brush-shaped active electrodes enabled completely non-invasive multi-channel recording of EEG and cortical CAEPs in unanesthetized horses. The use of active electrodes, which did not require skin preparation to lower skin-electrode impedance, made the electrode application process easier and more time-efficient compared to conventional passive electrodes. This reduction in preparation time allowed for the use of a greater number of electrodes while ensuring sufficient time for EEG recording. Specifically, our protocol required < 30 min to prepare a horse for EEG recording, even with a multi-electrode array of 12 electrodes, which provided greater scalp coverage than previously reported. Furthermore, the procedure did not require hair shaving, offering a significant advantage over previous methods that relied on passive electrodes. Our novel method enhances veterinary care, promotes research, and improves animal welfare by providing a means for evaluating brain functions and their disorders in alert horses and, potentially, other medium- to large-sized animals significant to humans, such as pigs and cattle.

To our knowledge, this is the first study to record CAEPs in horses, whether awake or sedated, although brainstem auditory evoked potentials under anesthesia have previously been reported (7, 8). Similar to the human CAEP, evident cerebral cortical responses were recorded in horses to the onset and offset of sounds, and candidate homologs of the human P1, N1, P2, and N2 components could be identified. Although their absolute latencies were shorter than those typical for humans, this finding is not surprising, as humans have the longest latencies for these components compared to other primate species (19, 20, 37). Whether these potential CAEP components in horses represent true functional homologs to human counterparts requires further research using more advanced neurophysiological and experimental techniques. Nevertheless, the existence of cortical auditory potentials analogous to human CAEPs provides an avenue to understand auditory processing and perception in an important domestic animal species, where most previous experiments on equine perception and cognition used the visual modality (1, 2).

The study employed a convenient method for determining the position of the vertex electrode (Cz), namely, the intersection of diagonals connecting the inner corner of one eye to the medial edge of the contralateral earlobe. Although this scalp location is unlikely to match the position of Cz as defined in human EEG, such a mismatch is difficult to avoid due to the challenges in identifying the referential landmarks for the 10-20 system (inion, nasion, and preauricular points) in the horse. Nevertheless, the Cz as determined by the current method apparently approximated the vertex in prior equine EEG/EP experiments (7–9, 13), although most of these studies did not describe the exact method for determining its position, making strict comparison difficult. The current method of determining the vertex position is hoped to establish a common ground for comparing electrode positions among different studies. Determining the locations of the other non-vertex electrodes remains an issue for future research.

## 5 Conclusion

In conclusion, this study described a novel method for non-invasively recording multi-channel EEG and evoked potentials from awake horses. The successful identification of potential equine homologs to human CAEPs demonstrated the feasibility and validity of this approach for investigating brain functions in horses. This paves the way for further applications, such as examining normal cognitive abilities, brain disorders, and ultimately furthering our understanding of the mind of this important domestic animal species.

## Data Availability

The raw data supporting the conclusions of this article will be made available by the authors, without undue reservation.
